# It Is Feasible to Produce Olive Oil in Temperate Humid Climate Regions

**DOI:** 10.3389/fpls.2019.01544

**Published:** 2019-11-27

**Authors:** Paula Conde-Innamorato, Mercedes Arias-Sibillotte, Juan José Villamil, Juliana Bruzzone, Yesica Bernaschina, Virginia Ferrari, Roberto Zoppolo, José Villamil, Carolina Leoni

**Affiliations:** ^1^ Instituto Nacional de Investigación Agropecuaria (INIA), Programa Nacional de Investigación en Producción Frutícola, Estación Experimental INIA Las Brujas, Canelones, Uruguay; ^2^ Unidad de Ecofisiología de Frutales, Departamento de Producción Vegetal, Facultad de Agronomía, Universidad de la República, Montevideo, Uruguay

**Keywords:** *Olea europaea* L., olive cultivars, phenological behavior, oil yield, productive efficiency, alternate bearing

## Abstract

Worldwide olive industry has expanded into new climatic regions outside the Mediterranean basin due to an increase in extra virgin olive oil demand posing new challenges. This is the case of Uruguay, South America, where the olive crop area reached 10,000 hectares in the last 15 years and is intended to the production of EVOO. Uruguay has a temperate humid climate with mean precipitations above 1,100 mm per year but unequally distributed, mild winters, and warm summers, with mean annual temperatures of 17.7°C. Different agroecological conditions require local knowledge to achieve good productivity whereby the objective of this work was to show the feasibility and potential of olive oil production under our climatic conditions. For this the agronomic performance of Arbequina, Barnea, Frantoio, Leccino, Manzanilla de Sevilla, and Picual cultivars was evaluated along 10 years of full production. Phenology behavior, vegetative growth rate, productive efficiency, alternate bearing, and oil yield were determined. Sprouting and flowering processes occur in a wide window within the annual cycle between the months of August to November with great interannual variation. More than 8 t/ha fruit yield and 40% oil yields in dry weight basis were obtained in promising cultivars. However, alternate bearing arose as the main production limiting factor, with ABI values greater than 0.60 for most cultivars. We conclude that olive oil production in humid climate regions is feasible and the most promising cultivars based on productive efficiency are Arbequina and Picual.

## Introduction

In the last decades, the worldwide olive industry had expanded into new climatic regions outside the Mediterranean basin due to an increase in extra virgin olive oil (EVOO) demand associated to its beneficial effects on human health (International Olive Council. IOC, 2015). Among these new regions, some of them have Mediterranean like climate—San Diego, USA and México (Ayerza and Sibbett, 2001) and La Rioja, Argentina (Rondanini et al., 2014)—whereas others have quite different temperature regimes, particularly mild winters resulting in less chilling hours for flowering during winter dormancy—Argentina, Australia (Aybar et al., 2015; Torres et al., 2017). Also, different humid regions are under olive cultivation, from wet hot summers, and cold dry winters—Wudu, China (Wang et al., 2018) to tropical—Queensland, Australia (Mailer et al., 2010) or temperate humid regions with high humidity, precipitation above 1,100 mm/year and moderate temperatures—Southern Brazil and Uruguay (Torres et al., 2017). In all of these new regions, EVOO is produced and particular olive profiles are obtained (Aguilera et al., 2005; Borges et al., 2017; Ellis and Gámbaro, 2018) showing the potential for EVOO expansion worldwide.

Oliviculture devoted to EVOO production is considered a new agricultural activity in Uruguay. However, olive introduction dates back to 1,780 along with the Spanish settlers (Pereira et al., 2018) and the first olive growing activity for virgin olive oil production started in the 1930s. Since 2002, modern orchards were planted for virgin olive oil production, increased from 500 to 9,000 hectares (Ackermann et al., 2018) with high technology olive oil mill. These new orchards were planted with certified material from Spain, Italy, Israel, and Argentina (Tous et al., 2005) being the main cultivar Arbequina (50%) followed by Picual, Coratina, Frantoio, Leccino, and Manzanilla de Sevilla (all adding up to around 40%). Uruguay is recognized by international olive oil sensory panels for its EVOO (Ellis and Gámbaro, 2018; ASOLUR, 2019). Currently, olive production accounts for 23% of the national area devoted to fruit crops, considering citrus, vineyards, and deciduous fruit trees, which evidences the importance of the olive in the fruit sector. Uruguay is the second smallest country in South America with 16.4 million hectares with agricultural aptitude and with little more than 3 million total inhabitants (Archivo General de la Nación, 2011; Instituto Nacional de Estadística. INE, 2011).

Modern olive plantations in Uruguay follow an intensive rainfed system within a range of 285–400 trees/ha, identified as system “S5” by IOC (International Olive Council. IOC, 2015). Less than 5% of the plantations are irrigated system “S6” (Rallo et al., 2013) although there are no official data. One of the reasons for not investing in irrigation in the productive systems is the annual pluviometry, generally greater than 1,100 mm per year, but highly variable among seasons and years. Due to the high frequency of periods of drought or pluviometric excesses in our conditions (Vaughan et al., 2017), the yield potentials of the productive systems are highly dependent on soil physical aptitude, topography, and associated soil management. The excess of rainfall at some given key stages of the vegetative—productive olive cycle (flowering—fruit set—ripening) generate different challenges for the development of olive trees in humid regions (Tous et al., 2005). According to the thermal requirements of the species proposed by Moriondo et al. (2008), Uruguay is an area suitable for the development of olive production. The average annual temperature is 17.7°C, varying from 19.8°C in the northwest zone to 16.6°C in the south coast. Isotherms have an incremental trend from the southeast to the northwest. The national averages of the annual extreme temperatures of the air present a maximum historical average of 22.6°C and a minimum average of 12.9°C (Castaño et al., 2011). The offer of winter cold varies between 500 and 1,000 chilling units, a factor that could be limiting for some deciduous fruit crops (Contarin and Curbelo, 1987; Severino et al., 2011). Average annual relative humidity between 70% and 78% associated to high annual rainfall, and moderate temperatures, favors the development of fungal diseases. The choice of cultivars with best behavior against pathogens is of utmost importance to achieve a sustainable orchard management (Leoni et al., 2018; Bernaschina et al., 2019).

There is extensive knowledge of the behavior of olive cultivars in traditional Mediterranean production areas, but not in regions with temperate-humid climate. Evaluation of a fruit species in a specific location requires the evaluation of its growth and development potential, precocity, and productive behavior (Klepo et al., 2013). Alternate bearing is a well-known condition in olive trees (Lavee, 2015), as well as its relationship with vigor and consequently with its productive efficiency. These conditions vary with cultivar but also with local agroecological conditions (Smith and Samach, 2013).

To identify limiting factors in olive production is necessary to start with cultivars phenological studies. After several years, the average and the overlap flowering period can be identified among cultivars in relation to climatic characteristics (Orlandi et al., 2005; Benlloch-González et al., 2018). Negative climatic events in non-traditional producing regions as Argentina and Chile, can result in null harvest, leading to regional alternate bearing processes (Cuevas et al., 1994; Goldschmidt, 2005; Beyá-Marshall and Fichet, 2017).

The objective of this work was to assess the feasibility and the potential of olive oil production in temperate-humid climate. For this, we evaluated the agronomic behavior of six cultivars characterizing their adaptability through phenology, growth rate, productive efficiency, alternate bearing index (ABI) and oil yield. This information is crucial for identifying promising cultivars in order to develop an integrated production strategy as well as to pursue technological development facing the expansion into new regions.

## Materials and Methods

### Experimental Sites and Plant Materials

Two experiments were established, one at INIA “Las Brujas” Experimental Station in Southern Uruguay (34°40’ S; 56°20’ W; altitude 21 m) and the other at INIA “Salto Grande” Experimental Station in Northern Uruguay (31°16’ S; 57°53’ W; altitude 41 m).

At INIA “Las Brujas” Experimental Station (LB), olives trees of Arbequina, Barnea, Frantoio, Leccino, Manzanilla de Sevilla, and Picual cultivars were planted in 2002 at a density of 416 trees per hectare (4 m between trees and 6 m between rows). A randomized complete block design with four replicates and three trees per experimental unit was used. The soil at this site has a fine textured A horizon, with a maximum depth of 20 cm, with 2.5% of organic matter and pH 6.5 corresponding to a Typic-Vertic Argiudolls soil according to the USDA classification (Durán et al., 2006). The presence of argillic B-horizons close to soil surface requires raised beds to increase soil volume to be explored by roots and to facilitate drainage.

At INIA “Salto Grande” Experimental Station (SG) olive trees of Arbequina, Frantoio, Manzanilla de Sevilla, and Picual cultivars were planted in 2003 at a density of 333 trees per hectare (5 m × 6 m). A randomized complete block design with six replicates was used and three trees per experimental unit. The soil at this site has a coarse textured A horizon, with a very good natural drainage and a maximum depth of 50 cm; therefore, there is no need for raised beds even though the strong textural differentiation between soil horizons. Soil natural fertility is poor, with less than 1.8% organic matter and a pH of 5.5. This type of soil corresponds to a Udifluvent soil according to the USDA classification (Durán et al., 2006).

Both experiments were managed with the same technological management for pruning, irrigation, and nutrition. Olive trees were trained as single-trunk vase, with three to four main branches. From 2002 to 2009, while the trees were filling its space within the row, pruning criteria was intended for training and thinning. From 2010 onwards, the control of tree height was included as a pruning criterion. Drip irrigation was installed at planting, arranged in simple rows with drippers spaced at 1 m and a flow of 4 l/h. After 5 years another identical line was added. Irrigation was applied to supply the water needed to replace 100% of the crop evapotranspiration (ETc) all throughout the irrigation season. Pest management was according to Integrate Pest Management guidelines (IOBC-WPRS, 2012) with control actions with horticultural oils and ant baits directed to *Saissetia oleae* and *Acromyrmex* and *Atta* ant species, respectively. For diseases, between four to six sprays per season with preventive cupric sprays complemented with other fungicides (QoIs, EBIs, dithiocarbamates) were applied for controlling Olive scab (*Venturia oleaginea*), Cercospora leaf spot (*Pseudocercospora cladosporioides*) and Anthracnose olive rot (*Colletotrichum* spp.) (**Supplementary Figure S1**).

### Climate

Meteorological data was obtained with an automatic weather station from 2007 to 2017 (data available at http://www.inia.uy/gras/Clima/Banco-datos-agroclimatico). Daily average mean temperature differed in almost 3°C between the two sites, with an average of 16.3°C and 19.0°C at LB and 19.0°C and 21.8°C at SG, for October and November, respectively. Also, the average effective precipitation was 109 and 105 mm per month in October and November at LB, and 134 and 142 mm at SG (**Supplementary Table S1**).

The offer of winter cold was measured with positive chill unit method (UTAH+) (Linsley-Noakes et al., 1994) from May 1^st^ to August 31^st^. The heat supply was measured with the GDH model in the period from July 1^st^ to December 31^st^ with a base temperature of 12.5°C (Galán et al., 2001).

### Phenological Parameters

Phenological records were made through visual assessments in individual plants based on the BBCH scale (Sanz-Cortés et al., 2002) along 10 years in which trees were between 5 and 15 years old. For each sampling date a unique predominant phenological state was registered for the set of trees of each cultivar in each site. The beginning of sprouting corresponds to the state BBCH 53, the beginning of flowering to BBCH 61 (10% flowers open), full bloom to BBCH 65 (at least 50% of flowers open) and end of flowering to BBCH 68 (majority of petals fallen or faded).

Sprouting and flowering windows were defined based on the first and the last date registered for each stage per cultivar between 2007 and 2017, and the dates were registered as the day of the year (DOY), starting on January 1^st^. At INIA Salto Grande, only the flowering stages were recorded. Flowering length days number was calculated by date calendar subtraction between states 61 and 68 of the BBCH scale.

### Productive Parameters

Fruit production (kg/tree) was measured per tree at both sites (n = 12 at LB, n = 18 at SG) and then fruit yield (t/ha) was calculated combining fruit production (kg/tree) and tree density (trees/ha) data. Canopy volume (CV, m^3^/tree) was measured only in the central tree of the plot during the first years (2007 to 2011) and in all trees of each plot from 2012 onwards. CV was calculated according to the ellipsoid volume method defined by three semi-axes (equation 1) with E_b_ and E_c_ as the North and East semi-axis of the tree, and E_a_ as the difference between tree height minus height of the first leaf and divided by two (Miranda-Fuentes et al., 2015). CV was not estimated for Barnea because the shape of the canopy does not fit the volume of an ellipsoid.

(1) $$\text{CV}=4/3\pi \;\times {\text{ E}}_{\text{a}}\;\times {\text{ E}}_{\text{b}}\;\times {\text{ E}}_{\text{c}}\;$$

For the period 2010–2017 and only for LB site, the productive efficiency (kg/m^3^ canopy) was estimated considering the years with high production (years “on”) and the ABI was calculated according to Monselise and Goldschmidt (1982) (equation 2).

(2) $$\text{ABI = (1/n-1) }\times \;\Sigma {\text{ (y}}_{n}-y_{n-1})/({\text{y}}_{n}+{\text{y}}_{n-1})$$

where y = yields in year n, with n varying from 1 to 8.

Oil content (%) was measured on a sample of 200 g of olives per cultivar, with three replicates (Shahidi, 2001). Each sample was grounded with a hammer mill and humidity determined at 105°C for 48 h, then the dried sample was grounded with a mortar and oil content analyzed in duplicate following the Soxhlet method.

Olives were collected only at “on” years (2012, 2014, 2016, and 2017 at INIA Las Brujas; and 2012, 2014, 2015, and 2017 at INIA Salto Grande) at fruit ripening index (FRI) 3 based on a 0–7 scale (Uceda and Frias, 1975) with 0: intense green skin, 3: reddish or purple skin in more than half of the fruit, 4: black skin and white pulp and 7: black skin and totally purple pulp. FRI was calculated as (equation 3):

(3) $$\text{FRI}=\Sigma ({\text{n}}_{i}*\text{I})/\text{N}$$

where n*_i_* = number of olives with i ripening level, I = ripening level going from 0 to 7, N = total number of olives evaluated (100 olives).

### Statistical Analysis

Most data were subjected to analysis of variance (ANOVA) and the interaction “cultivar **×** site” was studied. To highlight the productive potential of the cultivars for each site, an independent analysis per site is presented. For canopy development data from 2007 to 2010 was analyzed by a non-lineal regression following an exponential model (equation 4) and goodness of fit was estimated by Pearson correlation between measured and predicted values.

(4) CV = a ∗ e^(b ∗ tree age)

For the cumulative fruit yield a General Lineal Mixed Model was adjusted, considering cultivar and site and its interaction as fixed effect and site > block nested and block as a random factor. Tukey test at *p* ≤ 0.05 were calculated to separate means. The statistical program used was InfoStat version 2017 (Di Rienzo et al., 2017).

## Results

### Phenological Parameters

**Figure 1:**
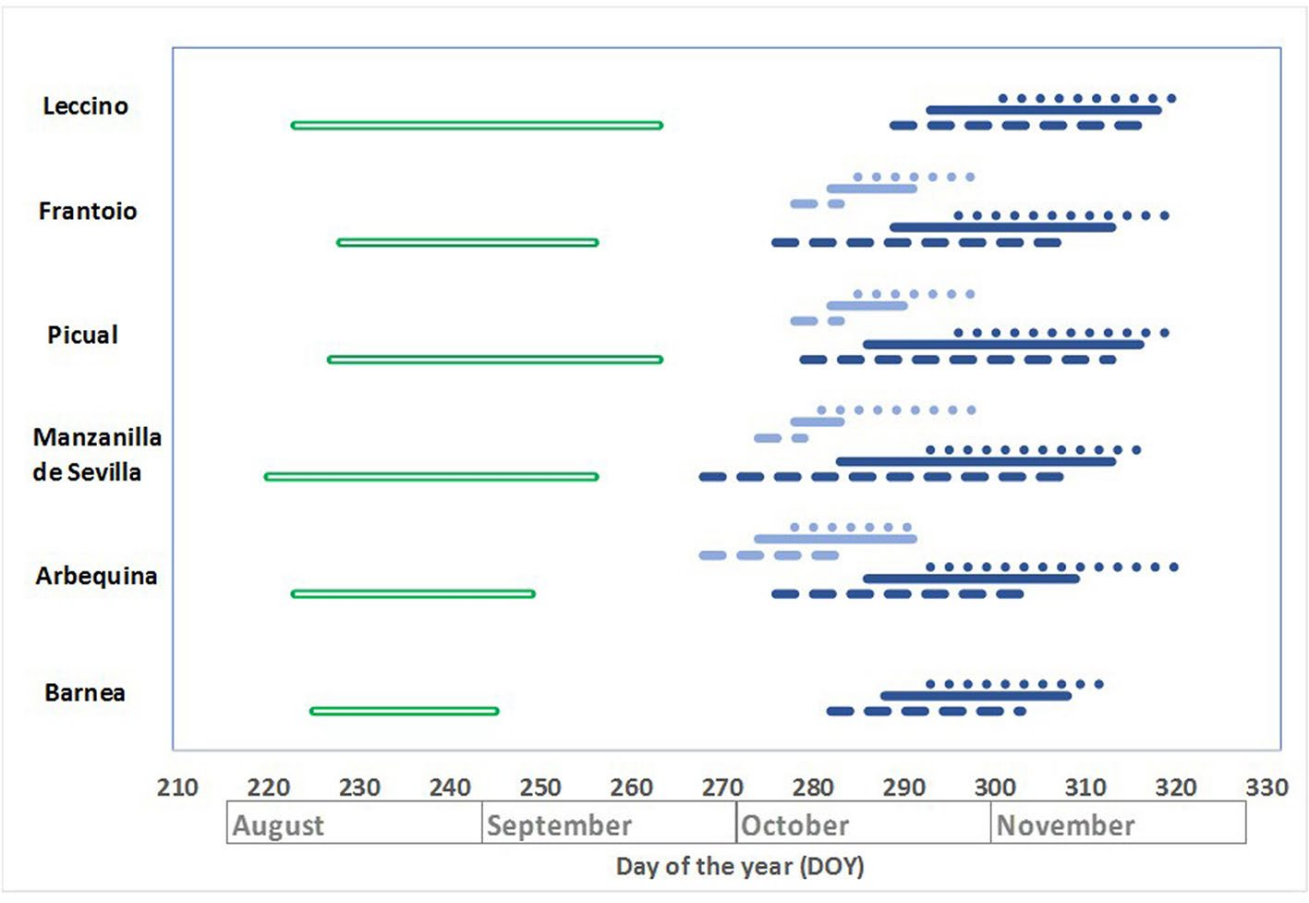
Lifespan of key phenological stages at INIA Las Brujas (LB) and INIA Salto Grande (SG), based on 10 years data. Beginning of sprouting, green completed double line; beginning of flowering, blue dashed line; beginning of full bloom, blue complete line; end of flowering, blue dotted lines. Light lines and dark lines correspond to SG and LB sites, respectively.

Flowering periods and sprouting (only for LB) were recorded along 10 years for six cultivars at LB and four at SG. Phenological data was analyzed following two different approaches: the lifespan in which the phenological phase occurred (**Figure 1**) and the length of the phenophases within each year (**Table 1**).

**Table 1:** Flowering length based on 10 years data (average with standard errors, minimum and maximum length) at INIA Las Brujas (LB) and INIA Salto Grande (SG).

Cultivar	LB			SG		
	Flowering length (days)	min. (days)	Max. (days)	Flowering length (days)	min. (days)	Max. (days)
Leccino	11.3 ± 1.2	7	14	–	–	–
Frantoio	14.2 ± 1.7	9	28	12.0 ± 3.2	7	18
Picual	13.5 ± 1.6	7	25	12.0 ± 3.2	7	18
Manzanilla de Sevilla	13.5 ± 1.9	7	29	11.8 ± 2.6	7	19
Arbequina	15.1 ± 1.8	7	28	11.3 ± 2.5	6	18
Barnea	12.1 ± 1.0	7	15	–	–	–

Sprouting windows started between the first week of August and the second week of September (**Figure 1**). The narrower variation in the beginning of sprouting at LB was registered for Barnea with a difference of 20 days among years, from 225 to 245 DOY. On the other hand, Leccino had the widest variation with a difference of 40 days, from 223 to 263 DOY.

Flowering windows from 10% flowers open (BBCH 61) to most petals fallen (BBCH 68) was highly variable among years. Considering all cultivars, the earliest date for BBCH 61 was 268 DOY in Manzanilla de Sevilla and the last date for BBCH 68 was 320 DOY in Arbequina. Therefore, the window for flowering in Southern Uruguay was 52 days, from the end of September to the end of November, for the 10 years evaluated.

The beginning of full bloom (BBCH 65) period at LB occurred between the second week of October and the second week of November, with a lifespan of 20 days in Barnea and 30 days in Manzanilla de Sevilla and Picual. At SG, the beginning of BBCH 65 occurred between the first and third week of October, with a lifespan of 5 days in Manzanilla de Sevilla and 17 days in Arbequina.

The average flowering length varied between 11.3 and 15.1 days at LB for Leccino and Arbequina, respectively, and between 11.3 and 12.0 days at SG for Arbequina and Frantoio, respectively. Despite cultivar nor site, the shortest flowering length was 7 days and the longest was 29 (**Table 1**).

The flowering behavior in two contrasting years regarding its thermal regimes was different (**Table 2**). In the warmest year (2017), the beginning of the flowering (BBCH 61) anticipated 15 and 18 days and the flowering length was 11 and 10 days longer respect to the coldest year (2016), for Arbequina and Frantoio, respectively.

**Table 2:** Chilling units (CHU) and heat supply (GDH) in the warmest and coldest years of the 10 years study at INIA Las Brujas and corresponding day of the year (DOY) for beginning and end of the flowering period, and flowering length for Arbequina and Frantoio cultivars.

Year	CHU^a^	GDH^b^	Cultivar	Beginning of flowering (DOY)	End of Flowering (DOY)	Flowering length (days)
2016	1353	691	Arbequina	291	308	17
			Frantoio	294	312	18
2017	926	756	Arbequina	276	304	28
			Frantoio	276	304	28
Historical mean^c^	1105	700				

^a^CHU (UTHA+) from May 1^st^ to August 31^st^; ^b^ GDH temperature over 12.5°C from July 1^st^ to December 31^st^; ^c^ Historical means of the period 1990 to 2017.

### Productive Parameters

**Figure 2:**
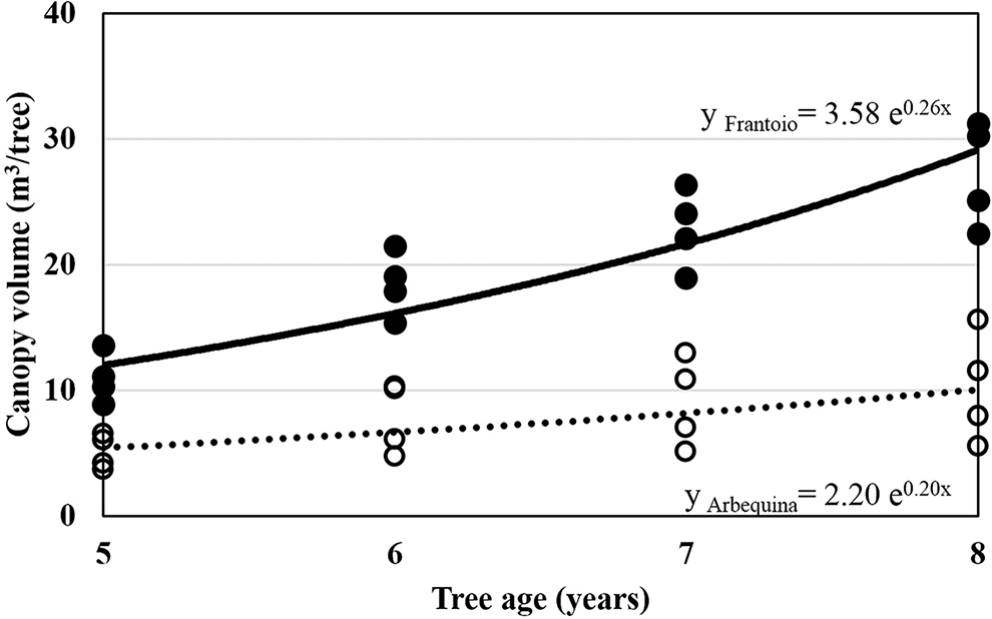
Progress of canopy volume from 2007 to 2010 of olive trees growing at INIA Las Brujas. Full circles correspond to Frantoio and empty circles correspond to Arbequina. The line represents the fitted exponential model of canopy volume.

The cultivars showed differences in canopy volume (CV), Frantoio presented the higher values and Arbequina the lower ones (**Table 3**, **Figure 2**). In 2011 with 9-year-old trees, because of pruning practices mainly to reduce tree height, no differences were found in CV. In 2016 with 14-year-old trees, despite pruning practices again Frantoio had the biggest tree size (CV: 39 m^3^/tree) and Arbequina the smallest one (CV: 26 m^3^/tree) (**Table 3**). In other words, in 2007 (5-year-old trees) Arbequina was 47% of Frantoio´s size, while at the end of the study period (14 year-old-trees) Arbequina was 66% of Frantoio´s size.

**Table 3:** Average canopy volume per tree for five cultivars for 5-, 9- and 14-year-old trees at INIA Las Brujas.

Cultivar	Canopy volume (m^3^/tree)					
	5-year-old trees		9-year-old trees		14-year-old trees	
Leccino	4.89	b	11.63	a	33.05	ab
Frantoio	10.95	a	15.25	a	39.07	a
Picual	6.84	ab	10.46	a	27.25	b
Manzanilla de Sevilla	5.28	b	11.98	a	28.46	b
Arbequina	5.11	b	8.74	a	25.88	b
Pr(>F)	0.004		0.124		< 0.0001	

Different letters in the column indicate significant differences (HSD Tukey p ≤ 0.05).

The progress of CV fitted an exponential model for the period 2007–2011. Arbequina had the lowest growth rate of change (0.20), estimated by parameter *b* of the exponential equation (equation 4) (**Table 4**, **Figure 2**).

**Table 4:** Estimated parameters *a* (intercept) and *b* (growth rate of change) of the exponential model for the progress of canopy volume CV = *a* * *e*^(*b* * tree age) for five cultivars from 2007 to 2010, at INIA Las Brujas.

Cultivar	Intercept (*a*)		Growth rate of change (*b*)		Goodness of fit
	estimate	Std. error	estimate	Std. error	
Leccino	1.09^+^^a^	0.60	0.33***	0.08	0.79
Frantoio	3.58**	1.01	0.26***	0.04	0.89
Picual	1.43 ns	1.03	0.30**	0.10	0.65
Manzanilla de Sevilla	1.60*	0.69	0.27***	0.06	0.80
Arbequina	2.20 ns	1.37	0.20*	0.09	0.53

^a^*** < 0.0001, ** < 0.001, * < 0.01, ^+^ < 0.05, ns: not significant.

**Figure 3:**
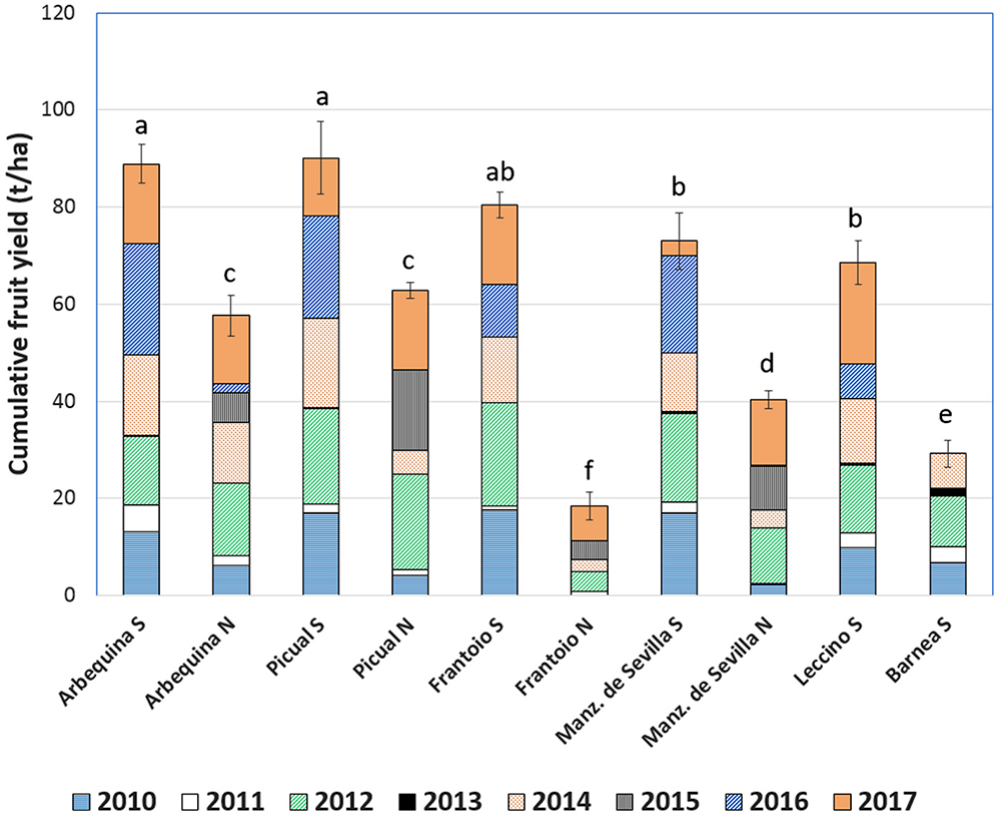
Cumulative fruit yield of six cultivars at INIA Las Brujas and four at INIA Salto Grande between 2010 and 2017. Bars with a common letter are not significantly different (HSD Tukey *p* ≤ 0.05).

Since no differences (*p* = 0.24) were found in CV/ha between LB and SG for each cultivar when the row space was filled, fruit yields were expressed in t/ha. Cumulative fruit yield presented significant differences among cultivars and regions (*p* < 0.0001) (**Figure 3**). Overall, cultivars produced more at LB than at SG. Arbequina (88 t/ha at LB, 58 t/ha at SG) and Picual (90 t/ha at LB, 63 t/ha at SG) presented the greatest cumulative yield in both sites. Frantoio was one of the most productive cultivars at LB with a cumulative fruit yield of 80 t/ha but at SG was the worst with18 t/ha. At LB site, Manzanilla de Sevilla and Leccino showed an intermediate fruit yield with 73 t/ha and 69 t/ha, respectively, whereas Barnea had a low fruit yield of 29 t/ha.

Alternate bearing behavior between the years “on” (high fruit production) followed by years “off” (low fruit production) was present in all cultivars (**Figure 3**). In 2013, the lowest fruit yield was recorded, being 0 t/ha for all cultivars except for Barnea which had a 1.6 t/ha fruit yield. The highest fruit yields where obtained in 2012 at LB and ranged between 10.4 t/ha to 21.3 t/ha for Barnea and Frantoio, respectively (Frantoio at SG was excluded in the range due to its extreme low fruit yield). At SG site, the alternate bearing behavior was typical along the years except for 2014 and 2015 when the yield was high in both years. At LB site occurred the same but in 2016 and 2017.

Arbequina and Picual had the highest productive efficiency (kg/m^3^ canopy) calculated for the “on” years, whereas Barnea had the lowest one (**Table 5**). All cultivars presented high ABI with values over 0.58. Barnea was the only cultivar that differed significantly for the ABI with the highest value (0.81).

**Table 5:** Productive efficiency calculated for the “on” years and alternate bearing index (ABI) for six cultivars at INIA Las Brujas.

Cultivar	Productive efficiency (kg/m^3^)^a^	ABI^b^
Leccino	1.21 bc^c^	0.63 b
Frantoio	1.08 bc	0.60 b
Picual	1.99 a	0.60 b
Manzanilla de Sevilla	1.69 ab	0.67 b
Arbequina	2.10 a	0.59 b
Barnea	0.84 c	0.81 a

^a^data only from “on” years, ^b^ data from the whole period, ^c^ different letters indicate significant differences within columns (HSD Tukey p ≤ 0.05).

For oil content, the interaction “cultivar **×** site” was not significant (*p* = 0.13 for dry weigh basis, and *p* = 0.43 for fresh weigh basis), whereas it was significant for average fruit yield (*p* < 0.001). Oil content in dry weight basis (DWB) and in fresh weight basis (FWB) were the highest in Frantoio and the lowest in Manzanilla de Sevilla in both sites. The average fruit yield (t/ha) per year was higher in Picual, Arbequina, and Frantoio in the LB site, followed by Manzanilla de Sevilla and Leccino and being the lowest in Barnea cultivar. In the SG site, Arbequina and Picual showed the higher yields, followed by Manzanilla de Sevilla and the lowest was for Frantoio cultivar (**Table 6**).

**Table 6:** Oil content in dry weight (DWB) and in fresh weight basis (FWB) and annual fruit yield for the six cultivars evaluated at INIA Las Brujas (LB) and four cultivars evaluated at INIA Salto Grande (SG).

Cultivar	Oil content^a^		Fruit Yield^b^
	DWB (%)	FWB (%)	Average (t/ha/year)
**LB** **^c^**			
Leccino	41.5 ab^d^	17.1 ab	8.6 b
Frantoio	46.6 a	22.9 a	10.1 ab
Picual	45.0 ab	17.0 ab	11.3 a
Manzanilla de Sevilla	36.8 b	13.1 b	9.1 b
Arbequina	43.0 ab	17.7 ab	11.1 a
Barnea	44.2 ab	22.0 a	5.0 c
			
**SG**			
Frantoio	46.3 a	22.5 a	2.3 c
Picual	39.4 b	14.8 b	7.9 a
Manzanilla de Sevilla	36.7 b	12.6 c	5.1 b
Arbequina	40.0 b	16.0 b	7.9 a

^a^data only from “on” years; ^b^data from the whole period; ^c^each region was analyzed separately; ^d^different letters in the column indicate significant differences (HSD Tukey p ≤ 0.05).

## Discussion

### It Is Feasible to Produce Olive Oil in Humid Temperate Climate

Productive behavior of adult trees from six olive cultivars along 10 years, demonstrates that it is feasible to produce olive oil in humid temperate climate. In our experimental conditions with irrigation, annual average fruit yields including years “on” and “off”, exceeded 8 t/ha with oil content above 36% DWB (**Table 6**). However, the interannual variability in thermal and rainfall regimes (Vaughan et al., 2017) affects regular production. Fruit and oil yields obtained were similar to those obtained in traditional Mediterranean basin regions (García et al., 2013; Gispert et al., 2013; Rallo et al., 2013; Díez et al., 2016; Marino et al., 2017) as well as in new olive growing regions (Tapia et al., 2009; Trentacoste et al., 2015; Beyá-Marshall and Fichet, 2017).

### Alternate Bearing as the Main Production Limiting Factor

Alternate bearing was the main productive constraint identified in our conditions, evidenced by ABI values above 0.60 (**Table 5**), 60% higher than those already reported for the same cultivars (Tapia et al., 2009). These ABI values imply fruit yield variations between 0 and 25 t/ha per year, similar to those mentioned by Lavee (2007). Despite the methodological approach of the present work was not intended to identify the main causes of alternate bearing, the synchronization observed among the cultivars in their expression of years “on” and “off” (**Figure 3**) allowed us to infer some of them. Alternate bearing could not be explained only by endogenous effects, for example, the growth regulators involved in the previous harvest, called the “biochemical memory” of fruit load (Haberman et al., 2017). But adverse meteorological effects in annual olive cultivars production arise as the driving force for alternate bearing, similarly to others fruit trees (Guitton et al., 2012).

It is widely reported that temperatures during autumn and winter, measured as chilling units and subsequently as heat supply affect both the beginning and the length of the phenophases (Nieddu et al., 2002; Galán et al., 2005; Orlandi et al., 2005). In our conditions, the window for the occurrence of sprouting ourred between early August and early September whereas for flowering it took place from mid-October to mid-November (**Figure 1**). This phenological behavior is in accordance with those reported for other regions of the Southern Hemisphere (Torres et al., 2017).

In “warm” years, characterized by winters with reduced contribution of chilling units and springs with early heat supply, the flowering period both anticipated and expanded. Similar processes have been reported by Rallo and Martin (1991); however, the interaction between cold requirements and heat requirements, to release dormancy in the olive trees is still unknown and hasn´t been determined as it has been in deciduous fruit species that have endodormancy (Pope et al., 2014).

There is evidence that winter cold correlates positively with the intensity of flowering (Rallo and Martin, 1991; Haberman et al., 2017; Rallo and Cuevas, 2017). Nevertheless, regardless of whether cold is necessary to induce flowering and/or to release reproductive bud dormancy, the number of chilling units required for each cultivar could be a limitation for our conditions with high interannual variability. In fact, at SG site where the cold supply of chilling units is lower than at LB site, Frantoio cultivar is not recommended due to its high cold requirements (Aybar et al., 2015).

An adequate pollination is required to ensure fruit set and therefore a good harvest, which also depends on weather conditions (Fernández, 2014). In our springs during the “flowering window”, although no extreme temperatures are present, average relative humidity above 70% in October and 60% in November as well as the probability of having 50% of the days with rain, affects negatively viable pollen-availability and therefore the fruit set. But also fruit set depends on pollen self-(in)compatibility of most olive cultivars (Marchese et al., 2016; Sánchez and Cuevas, 2018) where blooms synchronisms are crucial for our isolated plantation systems.

### Productive Efficiency Supports the Selection of Agroecologically Adapted Cultivars

Perennial polycarpic plants often show an inverse correlation between vegetative growth and the fruit production (Obeso, 2002; Smith and Samach, 2013). Productive efficiency is a key indicator to select cultivars for different orchard designs, mainly focusing on plant density (Rosati et al., 2013). We found the best productive efficiency in Picual and Arbequina, due to their high fruit production associated to relative low canopy volume. As in other olive production regions, Arbequina, based on productive efficiency among other traits like compact tree shape and thin branches, could potentially be selected for high density orchards (Tous et al., 1998; García et al., 2013; Connor et al., 2014; Trentacoste et al., 2015; Gomez Del Campo et al., 2017; Marino et al., 2017). However high-density plantations under humid-temperate conditions should be carefully evaluated because the risk of air-borne disease epidemics, specially anthracnose olive rot caused by *Colletotrichum* spp. On the other hand, Frantoio presents low productive efficiency due to its high vigor, but high fruit production and oil yield at LB site. Vegetative growth rates and canopy volumes reached in the first 5 years showed the potential vegetative development under our agroecological conditions, compromising early bearing as it was described in super high-density systems (Camposeo et al., 2008; Gomez Del Campo et al., 2017). These vigorous growths require differential pruning and nutrition techniques among cultivars in order to improve productive efficiency.

There were significant differences among evaluated cultivars, Picual and Arbequina are the most productive cultivars in both evaluated sites, reaching annual average fruit yields of 11 and 8 t/ha, at LB and SG, respectively. Although Barnea in Israel presents high productive potential (Dag et al., 2011), it is not suitable for our agroecological conditions due to its low fruit yield and high ABI. Other works that compare cultivars have also highlighted Arbequina, Picual, and Frantoio in diverse locations as the most productive ones (Tapia et al., 2009; Marino et al., 2017).

### Acceptable Oil Yields and Oil Contents

Oil contents were similar to those already reported in the Mediterranean basin, reinforcing the evidence that these are cultivar determined traits. The oil content in Frantoio was 46% DWB, as reported by Zeleke et al. (2012) while in Manzanilla de Sevilla only 37% DWB (**Table 6**), which associated with its great fruit weight makes it ideal for table olives (Rallo et al., 2018). Despite not measured in our study, virgin olive oils produced in Uruguay qualified as EVOO after evaluation of chemical and sensory properties (Ellis and Gámbaro, 2018) which reinforces the feasibility of EVOO production in temperate humid climates. Nevertheless, there is space for improving virgin oil yield, so studies aiming at understanding the reducing factors (weeds, diseases, soil pH) and limiting factors (water and nutrient supply) should be implemented.

### Disease Management Challenges Safety Olive Oil Production

Sanitary management is an important issue arising in new olive producing regions, since new or not problematic pests could compromise olive oil production (Lavee, 2014). Particularly in temperate humid regions, weather conditions (frequent rain events, elevated air relative humidity, air temperature above 20°C) are conducive to severe epidemic outbreaks. In Uruguay, unlike in the Mediterranean basin, pests are of secondary importance but can likely cause harm in new plantations along the first three-four years, as happens with the moth *Palpita forficifera*, with *Acromyrmex* and *Atta* ant species and with the scale *Saissetia oleae*. But undoubtedly diseases threaten olive production. Main fungal diseases are Olive scab (*Venturia oleaginea*), Cercospora leaf spot (*Pseudocercospora cladosporioides*), and Anthracnose olive rot (*Colletotrichum* spp.), with anthracnose being the riskiest one due to its direct damage on fruits and oil quality (Leoni et al., 2018). The conducive weather conditions for these diseases occur almost all year round, challenging orchard disease management.

In summary, olive production for virgin olive oil in humid temperate climate regions with high interannual variability is feasible. Potential fruit yields are subjected to appropriate cultivar selection and the expression of these yields will strongly depend on the adverse climatic conditions that could deepen the intrinsic alternate bearing. Nevertheless, is possible to rise productive efficiency and reduce ABI, but more local research on cultivar × environment × orchard management interactions is needed. The challenge is to develop an integrated strategy to improve current status and to address emerging problems.

## Data availability statement

The datasets generated for this study are available on request to the corresponding author.

## Author Contributions

JV and PC-I contributed to experimental design PC-I, JV, JB, MA-S, and CL contributed to experimentation PC-I, MA-S, and CL conducted the data analysis and writing of the manuscript. All authors contributed to manuscript revision, read, and approved the submitted version.

## Funding

The National Institute of Agricultural Research (Instituto Nacional de Investigación Agropecuaria – INIA, Uruguay) (Projects INIA HO08, INIA FR13) supported this work. We are grateful to David Bianchi, Richard Ashfield, and Jonathan Dávila for their collaboration in this project.

## Conflict of Interest

The authors declare that the research was conducted in the absence of any commercial or financial relationships that could be construed as a potential conflict of interest.

## Supplementary Material

Table S1 Average mean, maximum and minimum temperature, effective precipitation (Rain), precipitation days in the month, average relative humidity (RH) and evapotranspiration (Evap., Class A pan evaporation) from 2007 to 2017 and average historical data (1980–2009) at INIA Las Brujas and INIA Salto Grande, for October and November. Data was recorded from an automatic weather station (unless specified) and is available at http://www.inia.uy/gras/Clima/Banco-datos-agroclimatico.

Figure S1 Disease management scheme implementes at the experimental sites. OS = Olive Scab (*Venturia oleagina*), CLS = Cercospora leaf spot (*Pseudocercospora cladosporioides*), AOR = Antracnose olive rot (*Colletotrichum* spp.). *Optional treatment: based on previous season disease level ** QoIs, EBI or ditiocarbamates.
