# Supplementary material for: It Is Feasible to Produce Olive Oil in Temperate Humid Climate Regions
Source: Front Plant Sci. 2019 Nov 27;10:1544. doi: 10.3389/fpls.2019.01544 (PMC6893176; doi:10.3389/fpls.2019.01544)
Supplement: Table S1 — Average mean, maximum and minimum temperature, effective precipitation (Rain), precipitation days in the month, average relative humidity (RH) and evapotranspiration (Evap., Class A pan evaporation) from 2007 to 2017 and average historical data (1980–2009) at INIA Las Brujas and INIA Salto Grande, for October and November. Data was recorded from an automatic weather station (unless specified) and is available at http://www.inia.uy/gras/Clima/Banco-datos-agroclimatico. [file Table_1.pdf]

Table S1. Average mean, maximum and minimum temperature, effective precipitation (Rain), precipitation days in the month, average relative humidity (RH) and evapotranspiration (Evap., Class A pan evaporation) from 2007 to 2017 and average historical data (1980-2009) at INIA Las Brujas and INIA Salto Grande, for October and November. Data was recorded from an automatic weather station (unless specified) and is available at http://www.inia.uy/gras/Clima/Banco-datos-agroclimatico.

	**October**							**November**						
Year	Daily average temperature °C			Rain (mm)	Nº of days	RH (%)	Evap. (mm)	Daily average temperature °C			Rain (mm)	Nº of days	RH (%)	Evap. (mm)
	Mean	Max	Min					Mean	Max	Min				
**INIA Las Brujas**														
2007	17.2	22.8	11.9	131.4	12	77.3	120	17.1	23.1	10.3	58.6	12	71.6	175
2008	16.0	22.0	10.0	34.8	6	72.3	147	22.0	29.4	15.4	5.5	1	59.4	223
2009	14.8	20.7	8.8	134.0	9	74.7	115	19.1	24.4	14.1	126.4	13	75.5	141
2010	15.1	20.6	9.6	47.9	5	71.4	141	17.7	24.1	11.4	21.1	6	68.3	184
2011	15.3	20.3	10.6	69.4	7	71.9	133	19.9	26.5	13.4	115.3	8	62.8	212
2012	16.7	21.5	12.3	191.0	15	76.9	118	19.9	26.1	14.1	54.6	10	66.1	188
2013	16.0	22.1	9.8	8.7	6	70.5	157	18.8	24.1	13.7	158.9	11	72.3	170
2014	18.2	23.6	12.8	128.7	9	71.0	156	18.8	24.6	12.7	139.6	10	67.8	181
2015	14.2	19.0	9.9	59.4	9	72.4	130	18.1	24.1	12.2	72.3	6	65.9	186
2016	16.0	21.2	11.3	84.2	11	74.0	129	18.7	24.8	12.6	79.6	8	64.5	176
2017	16.1	21.5	10.5	80.1	11	71.1	143	17.7	24.2	11.3	34.3	5	63.9	220
1980-2009	16.3	22.1	10.5	109.3*	-	71.0	-	19.0	25.1	13.0	105.6*	-	69.0	-
**INIA Salto Grande**														
2008	18.0	23.5	13.4	3.0	7	73.6	155	23.8	29.9	14.7	0.0	4	64.2	330
2009	23.0	27.5	18.4	261.6	18	81.6	155	19.4	25.7	12.5	75.3	4	72.4	189
2010	17.5	24.1	10.3	52.1	6	72.1	183	21.1	28.2	13.2	44.5	4	68.0	240
2011	20.0	24.0	16.2	160.6	8	76.4	136	22.8	28.7	16.7	87.2	5	67.0	210
2012	19.5	23.8	14.8	251.2	15	83.6	140	23.0	28.9	17.0	54.1	3	75.0	240
2013	19.5	25.8	12.7	117.2	7	68.5	195	22.0	27.1	16.5	188.6	16	75.0	180
2014	21.1	26.6	15.4	139.9	10	75.8	161	22.0	27.9	16.0	150.5	10	71.0	180
2015	17.4	22.3	12.8	123.2	13	76.5	143	20.8	25.8	15.2	163.6	9	69.0	180
2016	19.0	24.4	13.6	170.3	15	71.9	152	21.6	28.0	14.9	62.9	7	65.0	210
2017	18.9	24.1	13.3	110.5	12	74.0	164	21.4	28.2	14.2	71.9	7	65.0	240
1980-2009	19.4	25.3	13.4	134.2	-	69.0	-	21.8	28.2	15.5	141.8	-	65.0	-

* data from Libertad agroclimatic station
