# Supplementary figures and images for: It Is Feasible to Produce Olive Oil in Temperate Humid Climate Regions
Source: Front Plant Sci. 2019 Nov 27;10:1544. doi: 10.3389/fpls.2019.01544 (PMC6893176; doi:10.3389/fpls.2019.01544)

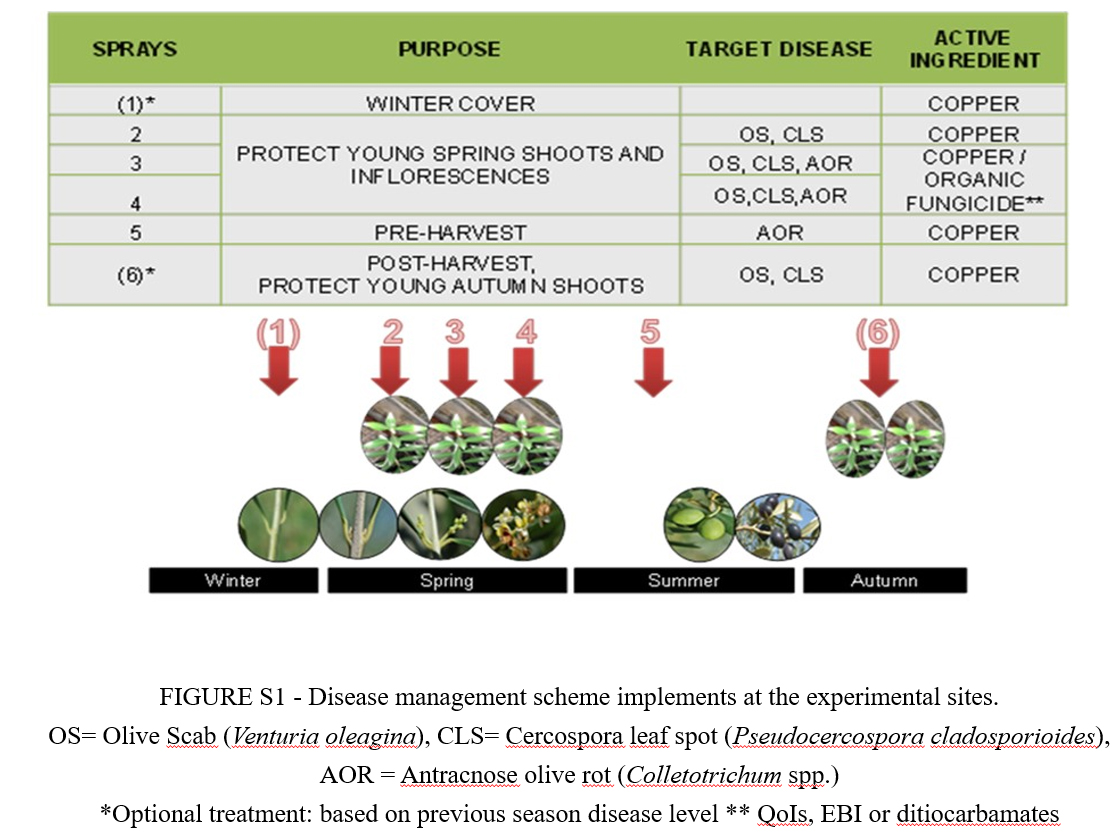

Supplement: Figure S1 — Disease management scheme implementes at the experimental sites. OS = Olive Scab (Venturia oleagina), CLS = Cercospora leaf spot (Pseudocercospora cladosporioides), AOR = Antracnose olive rot (Colletotrichum spp.). *Optional treatment: based on previous season disease level ** QoIs, EBI or ditiocarbamates. [file Image_1.jpg]
